# Leadership, job satisfaction and intention to leave among registered nurses in the North West and Free State provinces of South Africa

**DOI:** 10.4102/cur.v39i1.1585

**Published:** 2016-07-07

**Authors:** Jeremia S. Sojane, Hester C. Klopper, Siedine K. Coetzee

**Affiliations:** 1 School of Nursing Science, Potchefstroom Campus, North-West University, South Africa

## Abstract

**Background:**

The nurse leadership of a hospital is identified as the single most important aspect of the practice environment that impacts nurse outcomes. When nurses are satisfied with their jobs, they tend to remain with their employers and become more productive in their workplaces.

**Objectives:**

This study aimed to investigate the relationship between leadership, job satisfaction and intentions to leave among registered nurses (RNs) working in hospitals in the North West and Free State provinces of South Africa.

**Methods:**

A cross-sectional survey design was adopted. The population (*N* = 680) with the sample (*n* = 204) included RNs in medical–surgical units in both private and public hospitals in the two provinces. Data were collected using the RN4CAST questionnaire.

**Results:**

RNs were satisfied with the items pertaining to leadership except for praise and recognition (55.7%). They also indicated high levels of overall job satisfaction (70.5%) but were dissatisfied with wages (50%), study leave (40.9%) and opportunities for advancement (40.1%). Furthermore, 46.1% of the RNs intended to leave their current hospitals. The results indicated a relationship between leadership and job satisfaction (*r* = 0.47; *p* = 0.00) and between intention to leave and job satisfaction (*d* = 0.50).

**Conclusion:**

The nurse managers played a significant role influencing RN’s level of job satisfaction, while job satisfaction was highly correlated with intention to leave. The nurse leadership can improve job satisfaction by giving praise and recognition to the RNs for jobs well done, and RNs should be afforded the opportunity to advance their careers through further studies.

## Introduction and background

The nursing practice environment is defined as a comprehensive set of characteristics that enable ‘nurses to practice to their full potential’ (AACN 2002:298–300). According to Lake (2002), these characteristics include nurses participating in hospital affairs; staffing and resource adequacy; nursing foundation for quality care; nurse managers, ability, leadership and support of nurses; and collegial nurse–physician relationship.

International and national research has shown that nurses who work in hospitals with positive practice environments are less likely to report poor nurse outcomes, such as burnout, job dissatisfaction and turnover intent (Coetzee *et al*. 2013; Klopper *et al*. 2012; Zhang *et al*. 2014). Lambrou *et al*. (2014) conducted a systematic review to investigate nurse’s perception of their working environment in relation to job satisfaction. They found that the characteristic of the practice environment that had the most significant bearing on job satisfaction was the manager’s ability, leadership and support for nurses. Similarly, Vagharseyyedin (2016:107) conducted an integrative literature review on determinants of nurses’ organisational commitments and found that the four main reasons were personal characteristics and traits of nurses; leadership and management style and behaviour; perception of organisational context; and characteristics of job and work environment.

It is thus clear that nurse leadership and management affect nurses’ job satisfaction and organisational commitment. So, also, Mokoka, Oosthuizen and Ehlers (2010:8) reported that nurse managers influence the retention of registered nurses (RNs). Intention to leave is an intervening variable between actual turnover and job satisfaction, and it is therefore affected by similar individual and organisational factors (De Milt, Fitzpatrick & McNulty 2010). In South Africa, Coetzee *et al*. (2013) found that 51.3% of private and 59% of public medical–surgical nurses intend to leave their current hospital within the next year. The intention-to-leave rate among South African nurses was higher in comparison with nurses from the USA and Europe, except for Greece (Aiken *et al*. 2012).

Cummings *et al*. (2008:514) found that nurses would be more likely to remain in an employment situation if they experience autonomy, opportunities to participate in policy decision-making, support for innovation, supervisory support in managing conflict, a good working nurse–doctor relationship, visible leadership and if they have nurse managers who consult staff members. In South Africa, specifically, Minnaar and Selebi (2009:33) found that South African nurses stayed in particular workplaces if the nurse managers made them feel good about their jobs. Sveinsdottir, Ragnarsdottir and Blondal (2015:558) further researched this issue and found that nurses that were praised more often stated they had more opportunities to practise professionally, were more satisfied with their jobs, perceived the practice environment more positively and were more committed to the organisation.

The reviewed literature confirmed that nurse managers influenced nurses’ levels of job satisfaction and also nurses’ intentions to remain with an institution or to leave it.

### Problem statement

Practice environment is the single most important aspect influencing the well-being of nurses (Aiken *et al*. 2012; Coetzee *et al*. 2013). Within the practice environment, the characteristic of manager’s ability, leadership and support for nurses was the most influential on job satisfaction (Lambrou *et al*. 2014); so, also it was found that nurse managers influenced the retention of RNs (Mokoka *et al*. 2010:8). However, the precise relationship between leadership, job satisfaction and intentions to leave among RNs working in hospitals in the North West (NW) and Free State (FS) provinces remains unclear.

### Research question

The main research questions in this study were:

- What is the status of leadership, job satisfaction and intention to leave among RNs in hospitals in the NW and FS provinces?
- Is there a relationship between leadership, job satisfaction and intention to leave among RNs in hospitals in the NW and FS hospitals?

#### Hypothesis

In this study, the following hypothesis was tested:

H0: There is no relationship between nursing leadership, job satisfaction and intention to leave among RNs in hospitals in NW and FS provinces.

This study consequently explored the role of leadership and its relationship towards job satisfaction and RNs’ intentions to leave their positions in South Africa.

#### Research objectives

This study aimed to:

- Describe the status of leadership, job satisfaction and intention to leave among RNs in hospitals in the FS and NW provinces
- Investigate the relationship between leadership, job satisfaction and intentions to leave among RNs in hospitals in the FS and NW provinces.

#### Definitions of key concepts

*Leadership* is the relationship between the person in charge and the people who choose to follow (Kouzes & Posner 2002:4). A leader is a person that models the way, inspires a shared vision, challenges the process, enables other to act and encourages the heart (Kouzes & Posner 2007:14).

*Job satisfaction*, as defined by Robbins, Odendaal and Roodt (2003:72) and Lu, While and Barriball (2004:211), is the attitude or the feeling that an employee has towards various aspects of his/her job. The attitude develops when an employee feels positive about his/her working conditions and also when there are constructive responses from the organisation.

*Intention to leave* is described as the behavioural intention of an individual to voluntarily leave a profession or organisation (Terranova 2008:33). According to Bobko (2001), intention to leave refers to a decision made by an employee that is based on a continuum from initial thinking about leaving to implementing the actual behaviour of leaving.

A *registered nurse* is a person registered with the South African Nursing Council (SANC) as a nurse under article 16 of the *Nursing Act*, *No 33 of 2005*, as amended. The terms ‘registered nurse’ and ‘professional nurse’ are used as synonym.

## Research method and design

### Design

A quantitative, descriptive cross-sectional survey design was used to investigate the relationship between leadership, job satisfaction and RNs’ intention to leave hospitals in the FS and NW provinces. A cross-sectional survey design allowed the researcher to examine RNs’ experiences of leadership, job satisfaction and intention to leave simultaneously at one point of time (Burns & Grove 2009:695).

### Population and sample

This research focused on private and public hospitals in the FS and NW provinces. The population for this research study included all RNs in the medical and surgical wards of Level 1 three public hospitals in the FS province, Level 1 two hospitals in the NW province and three private hospitals from each of the selected private hospital groups in the FS and NW provinces. RNs in adult medical and surgical units participated in this study. An all-inclusive sample was used for RNs in medical and surgical units. A total of 680 questionnaires were distributed in the hospitals, and 204 RNs completed the questionnaire, amounting to a response rate of 33.3%. The total sample comprised 204 RNs (*n* = 204) from the FS and NW provinces, of whom 100 RNs worked in public hospitals and 104 RNs worked in private hospitals.

Inclusive criteria implied that RNs participating in this study had to be:

- RNs working in adult medical and surgical units of the selected hospitals;
- Proficient in English; as the RN4CAST questionnaires were available only in English.

### Data collection procedure

Data collection followed two processes to accommodate the dual (private and public) health-care system in South Africa. Data were collected using the self-administered questionnaire. In the private hospitals, a hospital employee (nurse) was appointed by the management of the hospital to manage data collection under supervision of a project manager. The project manager orientated the fieldworker for the RN4CAST programme and trained the fieldworker in the distribution and collection of the questionnaires. The fieldworker delivered the questionnaires to all nurses in the selected wards. Nurses returned the completed questionnaires to the fieldworker within one week. In the public hospitals, the project team delivered the questionnaires to all RNs in the selected wards on the morning of data collection and collected the completed surveys approximately six hours later.

The self-completion questionnaire was divided into four sections:

Section A enquired about your job, focusing on the practice environment of the RNs, and included the Practice Environment Scale of the Nurse Work Index (PES-NWI), questions related to job satisfaction, intention to leave and the Maslach Burnout Inventory (MBI).

Section B focused on quality and safety. In this section, RNs were asked to respond to issues related to safety and quality of care to patients delivered in their specific units.

Section C enquired about the nurses’ most recent shift at work in hospital. This section focused on questions related to the work schedules of RNs, nurse-to-patient ratios and details about the RNs’ most recent shifts.

Section D asked questions about ‘you the participant’. In this section, the demographic characteristics of RNs were explored, including questions related to age, gender and level of education (Sermeus *et al*. 2011).

In this study, the researcher only used the Nurse Manager’s Ability, Leadership and Support of Nurses subscales of the PES-NWI to measure leadership, overall job satisfaction and intention to leave (from section A of the questionnaire), and demographic data (from section D of the questionnaire).

### Data analysis

Both descriptive (means, standard deviations and percentages) and inferential statistics (Spearman’s rank order correlations, *t*-tests, Cronbach’s alpha and statistical significance) were used to analyse the data. In the RN4CAST programme, the raw information was entered and captured by computer software EpiData by the North-West University’s (NWU’s) Statistical Consultation Services (SCS). The data were analysed using the computer software statistical programme for social sciences (SPSS) version 16.0. (SPSS *Inc,* 2009). An analysis of the data was done in collaboration with a statistician from SCS.

## Reliability and validity

### Reliability

The leadership subscale of the revised PES-NWI consists of four items, namely: 1) supervisors support nurses; 2) a nurse manager is a good manager and a leader; 3) a nurse manager gives praise and recognition for a job well done; and 4) a nurse manager supports nurses in decision-making, even if it entails conflict with physicians. In international studies, Cronbach’s alpha coefficient for this subscale ranged between 0.63 and 0.84 (Bruyneel *et al*. 2009:205). In the national RN4CAST studies, the Cronbach’s alpha coefficient for this subscale was 0.86 (Klopper *et al*. 2012:690). In this study, the Cronbach’s alpha coefficient was 0.71, which means this could be accepted as being a reliable subscale to measure the variable of leadership.

Nurses’ overall job satisfaction was measured with a single item that ranged from 1 (very dissatisfied) to 4 (very satisfied). Published reliability coefficients for single-item overall job satisfaction ranged from 0.70 to 0.80 (Sermeus *et al*. 2011:4; Wanous, Reichers & Michael 1997). Nine specific aspects of job satisfaction (work schedule flexibility, opportunities for advancement, independence at work, professional status, wages, educational opportunities, annual leave, sick leave and study leave) were measured that ranged from 1 (very dissatisfied) to 4 (very satisfied). In addition, intent to leave was measured on a 2-point scale as ‘yes’ or ‘no’.

### Validity

The validity of the questionnaire was determined by an international pilot study done in Belgian hospitals during 2009. The predictive validity of the instrument used in the International Hospital Outcome Study (IHOS) was used in preparing the nurse survey questionnaire that was used in the RN4CAST project. The sample of 179 nurses had response rates that ranged from 67% to 79% across hospitals. Therefore, the IHOS Nurse Survey Questionnaire, used in the RN4CAST project, was a strong and psychometrically sound instrument (Bruyneel *et al*. 2009:202). The RN4CAST has been validated for allowing measurement, evaluation and comparison of nursing work environment factors that influence the work force (Bruyneel *et al*. 2009:203).

## Ethical considerations

The Ethics Committee of the North-West University approved the study (Certificate No: NWU-0015-08-S1). Thereafter, permission was requested from the three major private hospital groups to conduct the study in their hospitals; two of the major hospital groups granted permission. In the public hospitals, ethical clearance was received at national, provincial and district levels, as well as at each hospital. Respondents implied consent by completing and returning the questionnaires. The fieldworkers in the private hospitals, and project team in the public hospitals, informed each respondent that participation in the study was voluntary. No incentives were offered for participation. There were no potential risks for respondents. The questionnaires were coded and analysed at the SCS.

## Results

The research results will be discussed according to the major categories of the research instrument.

### Demographic and general information

The demographic information of the participants is presented in Table 1.

**TABLE 1:** Demographic information of participants (*n* = 204).

Variables	Frequency	(%)
**Gender**		
Female	193	94.6
Male	8	3.9
Not stated	3	1.5
**Age**		
20–29	24	12.4
30–39	48	24.9
40–49	67	34.7
50–59	45	23.3
60–69	9	4.7
**Level of education**		
Degree	39	19.1
Diploma	158	77.5
Missing	7	3.4
**Working in hospital**		
Full-time	195	95.6
Part-time	6	2.9
Missing	3	1.5
**Number of participants**		
Private hospital	104	51
Public hospital	100	49
**Years worked as RN**		
0–5	45	22.1
6–10	36	17.7
11–15	20	9.8
16–20	30	14.7
21–25	25	12.3
26–30	19	9.3
31–35	10	4.9
36–40	3	1.5
41–45	1	0.5
**Missing**	15	7.4
**Years worked in present hospital**		
0–5	73	35.8
6–10	44	21.6
11–15	32	15.7
16–20	18	8.8
21–25	10	4.9
26–30	5	2.5
31–35	1	0.5
36–40	2	1
**Missing**	19	9.3

*Source*: Authors’ own work

Table 1 shows that out of 204 RNs, 94% (*n* = 193) were females and 3.9% (*n* = 8) were males. This correlates with the SANC statistics indicating that FS had 7009 female RNs and 995 male RNs, while the NW province had 8145 female RNs and 1091 male RNs (SANC 2014). The majority of RNs (74%; *n* = 158) had diplomas in nursing. Of the 204 RNs, 34.7% (*n* = 67) were 40–49 years old. Of the RNs, 22.1% (*n* = 45) worked for 0–5 years, while 17.7% (*n* = 36) had worked for 6–10 years; 35.8% (*n* = 73) had worked for less than five years in the same hospital, and only 1% (*n* = 2) of RNs had worked for more than 36 years.

### Description of variables

Out of the 204 RNs, 71.0% (*n* = 142) agreed that their supervisors supported nurses, as shown in Table 2. Most respondents (75.7%; *n* = 153) agreed that their nurse managers were good leaders and good managers, and (65.8%; *n* = 131) agreed that their nurse manager will support the nurses’ decisions. However, with regard to nurse managers giving praise and recognition for a job well done, 55% (*n* = 112) disagreed with this item.

**TABLE 2:** Leadership (*n* = 204).

Item	Strongly disagree % (*n*)	Somewhat disagree % (*n*)	Somewhat agree % (*n*)	Strongly agree % (*n*)	Not stated	Mean	SD
A supervisory staff that is supportive of nurses	10 (20)	19 (38)	51.5 (103)	19.5 (39)	4	2.81	0.87
A nurse manager who is a good manager and leader	6.9 (14)	17.3 (35)	39.6 (80)	36.1 (73)	2	3.05	0.90
Praise and recog-nition for a job well done	18.9 (38)	36.8 (74)	31.8 (64)	12.4 (25)	3	2.38	0.93
A nurse manager who backs up the nursing staff in de-cision-making, even if the conflict is with the physician	14.1 (28)	20.1 (40)	36.7 (73)	29.1 (58)	5	2.81	1.01

*Source*: Authors’ own work

Leadership in medical and surgical units in the FS and NW provinces was reportedly effective, except that nurse managers did not praise nurses and did not recognise a job well done. However, the mean for the entire leadership subscale was 2.89 (SD = 0.63), which indicates that the requisite features of this subscale were present in the practice environments (Lake 2002).

Table 3 indicates that more respondents were satisfied 70.5% (*n* = 141) with their current jobs compared with the respondents who were dissatisfied 29.5% (*n* = 59). These findings were in line with national data, indicating that 32.2% of South African medical and surgical RNs experienced dissatisfaction with their jobs (Coetzee *et al*. 2013:162). Positive findings confirm that most participants (82.4%; *n* = 164) were satisfied with their independence at work and 81.4% (*n* = 162) were satisfied with their work schedule flexibility. Dissatisfaction was reported based on wages (50%; *n* = 101), opportunities for advancement (40.1%; *n* = 79) and educational opportunities (34.1%; *n* = 67).

**TABLE 3:** Job satisfaction (*n* = 204).

Item	Very dissatisfied % (*n*)	Little dissatisfied % (*n*)	Little satisfied % (*n*)	Very satisfied % (*n*)	Not stated	Mean	SD
**Job Satisfaction**							
How satisfied are you with your current job in this hospital?	12.5 (25)	17 (34)	55 (110)	15.5 (31)	4	2.74	0.87
**Personal advancement and reward**							
How satisfied are you with work schedule flexibility?	7 (14)	11.6 (23)	56.3 (112)	25.1 (50)	5	2.99	0.81
How satisfied are you with opportu-nity for advance-ment?	15.2 (30)	24.9 (49)	44.7 (88)	15.2 (30)	7	2.60	0.92
How satisfied are you with indepen-dence at work	7.0 (14)	10.6 (21)	47.2 (94)	35.2 (70)	5	3.11	0.86
How satisfied are you with profes-sional status?	5.7 (11)	14.4 (28)	49.5 (96)	30.4 (59)	10	3.05	0.82
How satisfied are you with wages?	30.7 (62)	19.3 (39)	39.6 (80)	10.4 (21)	2	2.30	1.02
How satisfied are you with educational opportunities?	20.8 (41)	13.3 (26)	43.1 (85)	22.8 (45)	7	2.68	1.05
**Leave**							
How satisfied are you with annual leaves?	7.5 (15)	13.1 (26)	46.2 (92)	33.2 (66)	5	3.05	0.88
How satisfied are you with sick leaves?	11.1 (22)	17.3 (34)	40.1 (79)	31.5 (62)	7	2.92	0.97
How satisfied are you with study leaves?	24.7 (49)	16.2 (32)	37.9 (75)	21.2 (42)	6	2.56	1.08
Satisfaction with nursing as a career of choice	8.3 (17)	9.8 (20)	31.9 (65)	47.1 (96)	6	3.21	0.94

*Source*: Authors’ own work

Most respondents were satisfied with their annual leave (79.4%; *n* = 158) and with their sick leave (71.6%; *n* = 141), but 40.9% (*n* = 81) were dissatisfied with their study leave.

Most respondents (81.3%; *n* = 161) were satisfied, while 18.9% (*n* = 37) were dissatisfied with nursing as a career of choice. This finding might explain the high incidence of job dissatisfaction because some respondents might have experienced job dissatisfaction because they did not enjoy nursing.

As many as 46.1% (*n* = 94) of the respondents indicated that, as a result of job dissatisfaction, they intended leaving their jobs in the current hospitals within the next year. Only 29.5% (*n* = 59) of the respondents were dissatisfied with their jobs and 18.1% (*n* = 37) were dissatisfied with their career choices. However, 46.1% (*n* = 94) of the respondents intended leaving their current employment positions within the next year as a result of job dissatisfaction.

Respondents, who intended to leave their places of employment within the next year as a result of job dissatisfaction, were asked what type of work they would seek. This item was measured on a three-point scale: ‘Nursing in another hospital, nursing, but not in a hospital or a non-nursing job’. Only 28% (*n* = 26) of these respondents would continue nursing in a hospital, 49.5% (*n* = 47) would continue nursing but not in a hospital setting and 22.4% (*n* = 21) would seek non-nursing employment. The finding indicates that 46.1% (*n* = 94) of the respondents intended leaving their current hospital jobs within the next year as a result of job dissatisfaction and only 28% (*n* = 26) would continue to work in hospitals.

Another question in relation to respondents’ intentions to leave their current jobs were asked, namely: ’If you were looking for another job, how easy do you think it would be for you to find an acceptable job in nursing?’ This item was measured on a four-point scale ranging from 1 (very difficult) to 4 (very easy). Most respondents (72.5%; *n* = 148) felt that it would be easy to find an acceptable job in nursing, while 22% (*n* = 45) felt that it would be difficult.

### Relationship between leadership and respondents’ job satisfaction

The correlation matrix in Table 4 presents the leadership subscale of the PES-NWI-revised in relation to overall job satisfaction and the nine aspects of job satisfaction. The *p*-value presents the statistical significance of the variables, whereas the correlation coefficient presents the magnitude, direction and strength of the relationship between the variables.

**TABLE 4:** Relationship between leadership and job satisfaction.

Variables		Leader-ship	Satisfaction with current job	Work schedule flexibility	Opportunities for advancement	Independence at work	Professional status	Wages	Educational opportunities	Annual leave	Sick leave	Study leave
**Leadership**	Correlation Coefficient	1.000	0.469**	0.348**	0.402**	0.359**	0.359**	0.335**	0.331**	0.347**	0.396**	0.338**
	Sig. (2-tailed)	-	0.000	0.000	0.000	0.000	0.000	0.000	0.000	0.000	0.000	0.000
	*n*	203	199	198	196	198	193	201	196	198	196	197
**Satisfaction with current job**	Correlation Coefficient	0.469**	1.000	0.416**	0.464**	0.438**	0.398**	0.444**	0.439**	0.317**	0.379**	0.409**
	Sig. (2-tailed)	0.000	-	0.000	0.000	0.000	0.000	0.000	0.000	0.000	0.000	0.000
	*n*	199	200	196	194	196	191	199	194	196	194	195
**Work schedule flexibility**	Correlation Coefficient	0.348**	0.416**	1.000	0.394**	0.481**	0.372**	0.254**	0.235**	0.350**	0.346**	0.299**
	Sig. (2-tailed)	0.000	0.000	-	0.000	0.000	0.000	0.000	0.001	0.000	0.000	0.000
	*n*	196	196	199	194	196	191	198	193	5	193	195
**Opportunities for advancement**	Correlation Coefficient	0.402**	0.464**	0.394**	1.000**	0.422**	0.461**	0.431**	0.654**	0.273**	0.254**	0.550**
	Sig. (2-tailed)	0.000	0.000	0.000	-	0.000	0.000	0.000	0.000	0.000	0.000	0.000
	*n*	196	194	194	197	193	189	197	192	194	192	194
**Independence at work**	Correlation Coefficient	0.359**	0.438**	0.481**	0.422**	1.000	0.681**	0.325**	0.364**	0.235**	0.338**	0.395**
	Sig. (2-tailed)	0.000	0.000	0.000	0.000	-	0.000	0.000	0.000	0.001	0.000	0.000
	*n*	198	196	196	193	199	193	198	193	195	193	194
**Professional status**	Correlation Coefficient	0.368**	0.398**	0.372**	0.461**	0.681**	1.000	0.404**	0.424**	0.384**	0.412**	0.490**
	Sig. (2-tailed)	0.000	0.000	0.000	0.000	0.000	-	0.000	0.000	0.000	0.000	0.000
	*n*	193	191	191	189	193	194	194	190	191	191	190
**Wages**	Correlation Coefficient	0.335**	0.444**	0.254**	0.431**	0.325**	0.404**	1.000	0.459**	0.374**	0.293**	0.367**
	Sig. (2-tailed)	0.000	0.000	0.000	0.000	0.000	0.000	-	0.000	0.000	0.000	0.000
	*n*	201	199	198	197	198	194	202	197	199	197	198
**Educational opportunities**	Correlation Coefficient	0.331**	0.439**	0.235**	0.654**	0.364**	0.424**	0.459**	1.000	0.418**	0.275**	0.685**
	Sig. (2-tailed)	0.000	0.000	0.001	0.000	0.000	0.000	0.000	-	0.000	0.000	0.000
	*n*	196	194	193	192	193	190	197	197	194	192	193
**Annual leave**	Correlation Coefficient	0.347**	0.317**	0.350**	0.273**	0.235**	0.384**	0.374**	0.418**	1.000	0.589**	0.444**
	Sig. (2-tailed)	0.003	0.000	0.000	0.000	0.001	0.000	0.000	0.000	-	0.000	0.000
	*n*	198	196	195	194	195	191	199	194	199	195	196
**Sick leave**	Correlation Coefficient	0.396**	0.379**	0.346**	0.254**	0.338**	0.412**	0.293**	0.275**	0.589**	1.000	0.466**
	Sig. (2-tailed)	0.000	0.000	0.000	0.000	0.000	0.000	0.000	0.000	0.000	-	0.000
	*n*	196	194	193	192	193	191	197	192	195	197	194
**Study leave**	Correlation Coefficient	0.338**	0.409**	0.299**	0.550**	0.395**	0.490**	0.367**	0.685**	0.444**	0.466**	1.000
	Sig. (2-tailed)	0.000	0.000	0.000	0.000	0.000	0.000	0.000	0.000	0.000	0.000	-
	*n*	197	195	195	194	194	190	198	193	196	194	198
	Sig. (2-tailed)	0.000	0.000	0.000	0.000	0.000	0.000	0.000	0.000	0.000	0.000	0.000
	*n*	200	198	197	195	197	192	200	195	197	1196	196
	Sig. (2-tailed)	0.000	0.000	0.002	0.001	0.000	0.000	0.006	0.000	0.000	0.000	0.000
	*n*	200	198	197	195	197	192	200	195	197	196	196

*Source*: Authors’ own work

Sig., significance;

** correlation is significant at the 0.01 level (2-tailed);

*, correlation is significant at the 0.05 level (2-tailed).

There was a large positive relationship between leadership and overall job satisfaction of RNs in their current hospitals (*r* = 0.47; *p* = 0.000). Furthermore, all nine aspects of job satisfaction showed a medium positive relationship (*r* = 0.33 – 0.44; *p* = 0.000).

Overall job satisfaction had a large positive relationship (*r* = 0.46; *p* = 0.000) with opportunities for advancement and a medium positive relationship (*r* = 0.32–0.44; *p* = 0.000) with all other job-related aspects.

### Relationship between nurses’ intention to leave, leadership and job satisfaction

The relationship between nurses’ intention to leave and the variables, leadership and job satisfaction, is visually presented by means of a 2×4 cross-tabulation. The reason for this is because intention to leave is measured on a two-point scale (nominal data) while leadership and job satisfaction are measured on a four-point scale (ordinal data).

In Table 5, the relationship between leadership items and intention to leave is presented with percentages, frequency (in brackets) and statistical significance.

**TABLE 5:** Relationship between leadership and intention to leave (*n* = 204).

Item	Intention to leave Response (*n*)	Strongly disagree % (*n*)	Somewhat disagree % (*n*)	Somewhat agree % (*n*)	Strongly agree % (*n*)	*p*-value
A supervisory staff that is supportive of nurses	1 = Yes (91)	15.4 (14)	22.0 (20)	45.0 (41)	17.6 (16)	0.074
	2 = No (107)	5.6 (6)	16.8 (18)	57.0 (61)	20.6 (22)	
A nurse manager who is a good manager and a leader	1 = Yes (92)	12.0 (11)	20.7 (19)	43.5 (40)	23.8 (22)	0.003
	2 = No (108)	2.8 (3)	14.8 (16)	37.0 (40)	45.4 (49)	
Praise and recognition for a job well done	1 = Yes (92)	33.7 (31)	29.3 (27)	31.5 (29)	5.5 (5)	0.000
	2 = No (108)	6.5 (7)	42.6 (46)	32.4 (35)	18.5 (20)	
A nurse manager who backs up the nursing staff in decision-making, even if the conflict is with a physician	1 = Yes (92)	21.7 (20)	25.0 (23)	33.7 (31)	19.6 (18)	0.002
	2 = No (106)	7.6 (8)	16.0 (17)	39.6 (42)	36.8 (39)	

*Source*: Authors’ own work

There is a small practical significant difference (*d* = 0.19) between the intention to leave and a supervisor who supported nurses. Table 5 indicates that RNs who intended to leave their current hospitals were more likely to disagree (37.4%; *n* = 34) that supervisors supported nurses, compared with RNs who intended to stay (22.4%; *n* = 24).

There is a medium practical significant difference (*d* = 0.26) between intention to leave and a nurse manager who was reportedly a good manager and leader. Table 5 demonstrates that RNs who intended leaving their current hospitals were more likely to disagree (32.7%; *n* = 30) that their nurse managers were good managers and leaders, compared with RNs who intended to stay (17.6%; *n* = 19).

There was a medium practical significant difference (*d* = 0.38) between intention to leave, and praise and recognition for a job well done. Table 5 shows RNs who intended to leave were more likely to disagree (63.0%; *n* = 58) that they received praise and recognition for a job well done than RNs who intended to stay (49.1%; *n* = 53).

There is a medium practical significant difference (*d* = 0.27) between intention to leave and a nurse manager who backed up the nurses’ decision-making, even if there was conflict with physicians. RNs who intended to leave were more likely to disagree (46.7%; *n* = 43) that their nurse manager supported them in decision-making, even if the conflict was with the physician, compared with RNs who intended to stay (23.6%; *n* = 25).

Table 6 portrays the relationship between RNs’ intention to leave and overall job satisfaction.

**TABLE 6:** Relationship between overall job satisfaction and intention to leave (*n* = 204).

Item	Intention to leave Response (*n*)	Very dissatisfied % (*n*)	Little dissatisfied % (*n*)	Little satisfied % (*n*)	Very satisfied % (*n*)	*p-value*
How satisfied are you with your current job in this hospital?	1 = Yes (93)	21.5 (20)	29 (27)	48.4 (45)	1.1 (1)	0.000
	2 = No (106)	4.7 (5)	6.6 (7)	61.3 (65)	27.4 (29)	
Opportunities for advancement	1 = Yes (92)	26.1 (24)	33.7 (31)	34.8 (32)	5.4 (5)	0.000
	2 = No (104)	5.8 (6)	17.3 (18)	52.9 (55)	24.0 (25)	
Wages	1 = Yes (94)	51.0 (48)	16.0 (15)	29.8 (28)	3.2 (3)	0.000
	2 = No (107)	13.1 (14)	21.5 (23)	48.6 (52)	16.8 (18)	
Sick leave	1 = Yes (89)	22.5 (20)	19.1 (17)	42.7 (38)	15.7 (14)	0.000
	2 = No (107)	1.9 (2)	15.9 (17)	38.3 (41)	43.9 (47)	
Study leave	1 = Yes (92)	40.2 (37)	15.2 (14)	33.7 (31)	10.9 (10)	0.000
	2 = No (105)	11.4 (12)	17.2 (18)	41.9 (44)	29.5 (31)	
Professional status	1 = Yes (91)	9.9 (9)	23.1 (21)	52.7 (48)	14.3 (13)	0.000
	2 = No (102)	2.0 (2)	6.8 (7)	46.1 (47)	45.1 (46)	
Independence at work	1 = Yes (93)	10.8 (10)	20.4 (19)	48.4 (45)	20.4 (19)	0.000
	2 = No (93)	3.8 (4)	1.9 (2)	45.7 (48)	48.6 (51)	
Work schedule flexibility	1 = Yes (92)	14.1 (13)	15.2 (14)	56.5 (52)	14.1 (13)	0.000
	2 = No (106)	0.9 (1)	8.5 (9)	56.6 (60)	34.0 (36)	
Educational opportunities	1 = Yes (92)	33.7 (31)	16.3 (15)	33.7 (31)	16.3 (15)	0.000
	2 = No (104)	9.6 (10)	10.6 (11)	51.0 (53)	28.8 (30)	
Annual leave	1 = Yes (91)	13.1 (12)	18.7 (17)	47.3 (43)	20.9 (19)	0.000
	2 = No (107)	2.8 (3)	8.4 (9)	44.9 (48)	43.9 (47)	

*Source*: Authors’ own work

There is a large practical significant relationship between RNs’ intention to leave and their overall job satisfaction (*d* = 0.50).

This is presented in Table 6 which shows that RNs who intended to leave their current hospitals were more dissatisfied (50.5%; *n* = 47) with their current jobs than RNs who intended to stay (11.3%; *n* = 12).

There was a medium practical significant difference between intention to leave and opportunities for advancement (*d* = 0.41). Table 6 shows that RNs who intended to leave their current hospitals were more dissatisfied (59.8%; *n* = 55) with the opportunities for advancement compared with RNs (23.1%; *n* = 24) who had no intention to leave their current hospitals.

There was a medium practical significant difference between intention to leave and independence at work (*d* = 0.39). RNs who intended to leave their current hospitals were more dissatisfied (31.2%; *n* = 29). with independence at work than RNs who intended to stay (5.7%; *n* = 6).

There was a medium practical significant difference between intention to leave and wages (*d* = 0.43). Table 6 indicates that RNs who intended to leave their current hospitals were more dissatisfied (67%; *n* = 63) with wages compared with RNs who intended to stay (34.6%; *n* = 37) at their current hospital.

There was a medium practical significant difference (*d* = 0.33) between intention to leave and educational opportunities. Table 6 shows that RNs who intended to leave their current hospitals were more dissatisfied (50%; *n* = 46) with educational opportunities compared with RNs who intended to stay (20.2%; *n* = 21).

There was a medium practical significant difference (*d* = 0.31) between intention to leave and satisfaction with annual leaves. Table 6 shows that RNs who intended to leave their current hospitals were more dissatisfied (31.8%; *n* = 29) with annual leaves compared with RNs who intended to stay (11.2%; *n* = 12).

There was a medium practical significant difference between intention to leave and sick leave, evidenced by a *d*-value of 0.40. As shown in Table 6, RNs who intended to leave were more dissatisfied (41.6%; *n* = 37) with sick leaves compared with RNs who intended to stay (17.8%; *n* = 19) at their current hospitals.

There was a medium practical significant difference (*d* = 0.36) between intention to leave and satisfaction with study leave. Table 6 shows that RNs who intended to leave their current hospitals were more dissatisfied (55.4%; *n* = 51) with study leaves compared with RNs who intended to stay (28.6%; *n* = 30).

## Discussion

Findings show that RNs in this study were mostly females (94.6%), diploma-prepared (77.5%) and permanently employed (95.6%). The majority of the respondents had 0–5 years of experience (35.8%). Pillay (2009:39) found that more young RNs intended to leave their current working areas after five years of working. This is especially in the public sector and more rural provinces.

The RNs were satisfied with the leadership of their nurse managers in their hospitals, except that their managers did not praise them and did not recognise a job well done. Of the RNs who intended leaving their hospitals, 63% (*n* = 58) were more likely to disagree that they received praise and recognition compared with those who intended to stay. According to Tourangeau and Cranley (2006:505), RNs’ satisfaction with praise and recognition received at work determined whether they wanted to remain employed in the same hospital. In the study by Oosthuizen and Ehlers (2007:21), 63.3% of RNs considered leaving the country because of lack of recognition.

In this study, most RNs (79%; *n* = 161) were satisfied with nursing as a career of choice, and 70.5% (*n* = 141) of the respondents were satisfied with their jobs, while 29.5% (*n* = 59) of the respondents were dissatisfied. Selebi and Minnaar (2007:56) reported that the overall job satisfaction of RNs in the Gauteng province was at a low level of 35%. The results of the current study thus showed a higher level of job satisfaction than other similar studies.

RNs in the FS and NW provinces in surgical and medical wards were satisfied with some aspects of their jobs. More prominent aspects included independence at work (82.4%; *n* = 164), work schedule flexibility (81.4%; *n* = 162), professional status (79.9%; *n* = 155), annual leave (79.4%; *n* = 158), sick leave (71.6%; *n* = 141) and educational opportunities (65.5%; *n* = 130). However, RNs were dissatisfied with opportunities for advancement (59.9%; *n* = 118), study leave (59.1%; *n* = 117) and wages (50%; *n* = 101). These findings showed that RNs wanted to advance in their careers, and if there is no possibility for advancement they might consider leaving their current hospitals within the next year.

RNs, who were dissatisfied with their wages (50%; *n* = 101), were more likely to intend to leave their current hospitals within the next year. In the study by Selebi and Minnaar (2007:57) in the Gauteng province, 96.6% of job dissatisfaction concerned wages. Wages influenced RNs to consider leaving their country to work in foreign countries, as they were unable to maintain a certain expected standard of living in South Africa (Oosthuizen & Ehlers 2007:21).

Of the current study’s respondents, 46.1% (*n* = 94) intended leaving their current hospitals within the next year. These rates are higher than a study conducted by Pillay (2009:39) in South Africa reporting that 34.8% of RNs intended to leave their hospitals within the next 5 years, because they were dissatisfied with wages, workload, career development and resources available to them, especially in public hospitals. These results were, however, lower than those of the national findings, which showed that over half of medical–surgical nurses intended leaving their jobs within a year, 51% in private hospitals and 59% in public hospitals (Coetzee *et al*. 2013:169).

There was a large-to-medium positive relationship between leadership and job satisfaction. According to Amadeo (2008:62), the leadership style in health-care settings affects job satisfaction of RNs.

There was a medium-to-small practical difference between nurses’ intention to leave and leadership, with those intending to leave being more dissatisfied with leadership compared with those who intended to stay. There was a large-to-medium practical difference between nurses’ intention to leave and job satisfaction, with those intending to leave being more dissatisfied with their jobs than those intending to stay. According to Lu *et al*. (2004:222), the current worldwide shortage of RNs highlights the necessity of understanding the impact and interrelationships between nursing leadership, job satisfaction and nurses’ intention to leave their workplaces.

Thus, the null hypothesis that there is no significant relationship between leadership, job satisfaction and intention to leave among RNs in the hospitals in the FS and NW provinces can be rejected.

## Limitations of the study

Data were collected only in the medical and surgical wards in the FS and NW provinces in both private and public hospitals. In the public hospitals, only Level 3 hospitals and a Level 2 hospital (that was preparing to become a Level 3 hospital) were included in the study, and consequently results may not be generalised to other levels and other provinces.

## Conclusions

The study indicated that 46.1% of the respondents intended to leave their current positions, of whom only 28.1% would continue to work in hospitals. This is perplexing, as nurses were generally satisfied with the items of leadership except for the praise and recognition aspects. They were also satisfied with professional status and leave, except for wages, opportunities for advancement, educational opportunities and study leave. This signifies that RNs should receive praise and recognition from their leaders and also be afforded the opportunity to advance their careers through further studies.

In the context of this study, RNs in the medical and surgical units showed that there was effective leadership and a high level of job satisfaction but also a high level of intention to leave. The study contributed to the current knowledge base of nursing in South Africa. To that end, the aim of the study was achieved. It is critical to question why South African RNs in the medical and surgical units intended to leave their current workplaces, although they had effective leadership and high levels of job satisfaction.

## Recommendations

Based on the conclusions of the study, it is recommended that nurse leaders should practise and emphasise praising and recognising the RNs’ work to create positive work environments. There should be in-service training to empower managers and nurse leaders in the recognition and praise of their staff. Furthermore, it is recommended that management of health and labour departments should reconsider the wages of the RNs in both public and private hospitals to keep the present RNs and to attract new RNs to the field, and also educational opportunities and study leaves should be revised so that RNs can advance their qualifications and have more opportunities for advancement. Nurse leaders and RNs should be trained in strategies to improve their practice environment and should be taught coping skills in order to ensure a higher level of job satisfaction. It is recommended that further qualitative research should be done to find detailed information about job dissatisfaction and RNs’ intention to leave. A policy should be developed to ensure that RNs are trained for leadership positions before they assume leadership duties besides doing management courses. It is necessary to consult and invite bedside nurses when developing hospital or ward policies.
